# Supplementary material for: Non-uniform distribution of myosin-mediated forces governs red blood cell membrane curvature through tension modulation
Source: PLoS Comput Biol. 2020 May 26;16(5):e1007890. doi: 10.1371/journal.pcbi.1007890 (PMC7274484; doi:10.1371/journal.pcbi.1007890)
Supplement: S1 Fig — (PDF) [file pcbi.1007890.s001.pdf]

Supplementary material for “Non-uniform distribution of myosin-mediated forces governs red blood cell membrane curvature through tension modulation”

H. Alimohamadi[1], A.S. Smith[2], R.B. Nowak[2], V.M. Fowler[2,3] and P. Rangamani[1]

[1]Department of Mechanical and Aerospace Engineering, University of California San Diego, California, United states of America
[2]Department of Molecular Medicine, The Scripps Research Institute, La Jolla, California, United states of America
[3]Department of Biological Sciences, University of Delaware, Newark, Delaware, Unites States of America

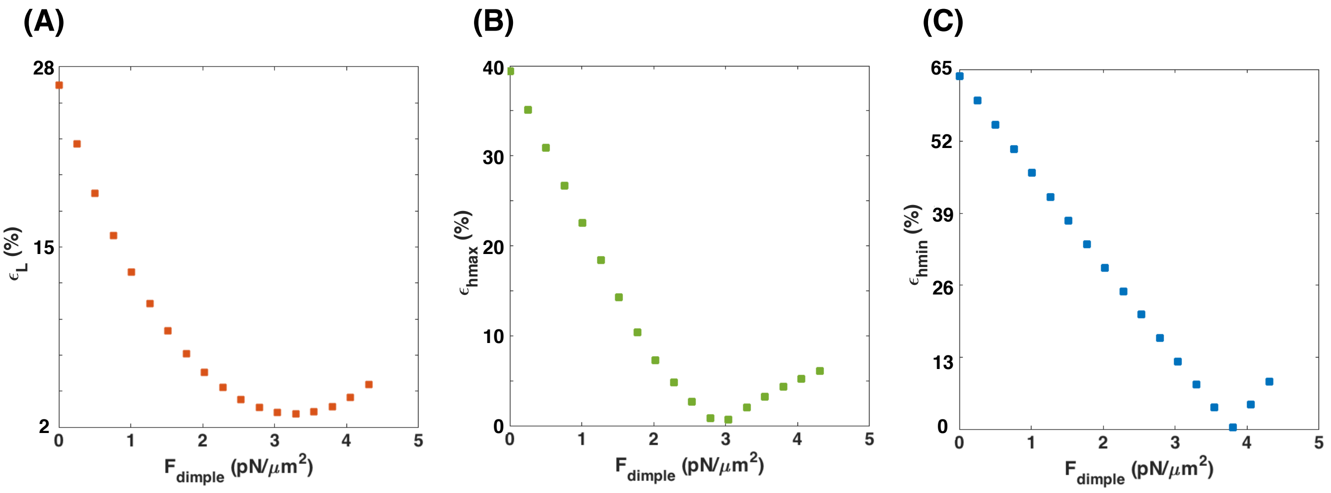

Figure S1: The error in the characteristic lengths is a nonlinear function of dimple force density (F_{dimple}). (A) Calculated error in the maximum length of the simulated RBC (ϵ_L) as a function of F_{dimple}. (B) Calculated error in the maximum height of the rim of the simulated RBC (ϵ_{hmax}) as a function of F_{dimple}. (C) Calculated error in the minimum height of the dimple of the simulated RBC (ϵ_{hmin}) as a function of F_{dimple}. In all three graphs, with increasing F_{dimple} from zero, initially the error decreases about an order of magnitude and attains a relative minimum. Any further increase in F_{dimple} toward the large dimple force density ($F_{dimple} > 4\mathrm{pN}/\mu\mathrm{m}^2$) leads to a larger error in all characteristic lengths.
