# Supplementary material for: Non-uniform distribution of myosin-mediated forces governs red blood cell membrane curvature through tension modulation
Source: PLoS Comput Biol. 2020 May 26;16(5):e1007890. doi: 10.1371/journal.pcbi.1007890 (PMC7274484; doi:10.1371/journal.pcbi.1007890)
Supplement: S2 Fig — (PDF) [file pcbi.1007890.s002.pdf]

Supplementary material for “Non-uniform distribution of myosin-mediated forces governs red blood cell membrane curvature through tension modulation”

H. Alimohamadi[1], A.S. Smith[2], R.B. Nowak[2], V.M. Fowler[2,3] and P. Rangamani[1]

[1]Department of Mechanical and Aerospace Engineering, University of California San Diego, California, United states of America
[2]Department of Molecular Medicine, The Scripps Research Institute, La Jolla, California, United states of America
[3]Department of Biological Sciences, University of Delaware, Newark, Delaware, Unites States of America

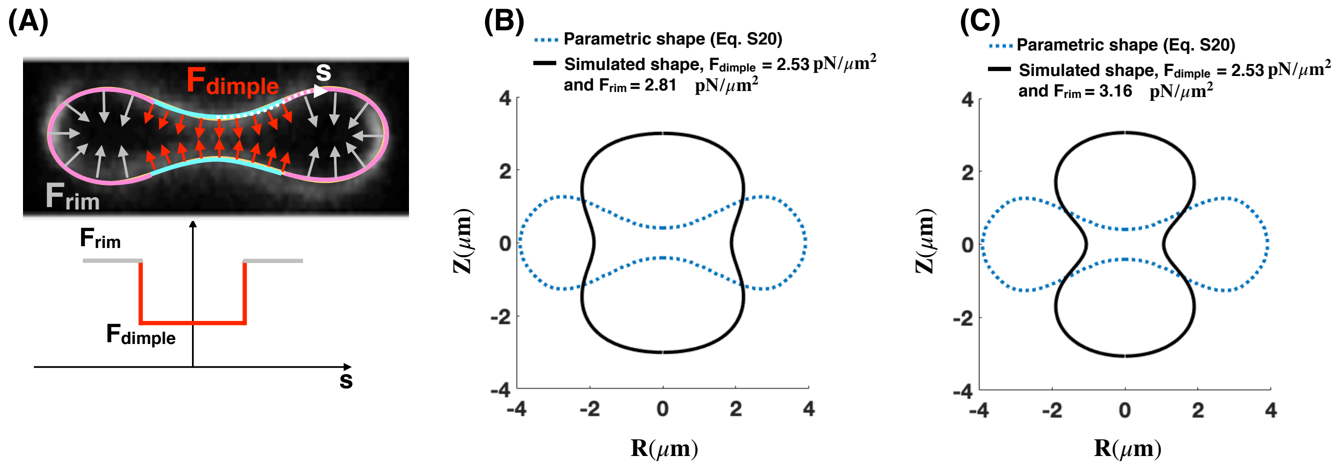

Figure S2: Applying a large pulling force at the rim region causes a very large shape error. (A, upper) Schematic of a biconcave RBC with a small force per unit area (red arrows) at the dimple and a large force per unit area (gray arrows) at the rim section. (A, lower) The applied force along the membrane as a function of the arclength. (B) A comparison between the simulated shape of RBC with F_{dimple} = 2.53 $\mathrm{pN}/\mu\mathrm{m}^2$ and F_{rim} = 2.81 $\mathrm{pN}/\mu\mathrm{m}^2$ and the parametric shape of an RBC. Having a larger force density along the rim region deviates the simulated shape from a biconcave to a peanut- shape geometry. Therefore, the calculated shape error becomes significantly large ($\epsilon_{total} \sim 52\%$). (C) The parametric shape of an RBC versus the shape that obtained from the simulation with F_{dimple} = 2.53 $\mathrm{pN}/\mu\mathrm{m}^2$ and F_{rim} = 3.16 $\mathrm{pN}/\mu\mathrm{m}^2$. As the rim force density increases, we found a much larger shape error in the simulated geometry ($\epsilon_{total} \sim 91\%$).
