# Supplementary material for: Non-uniform distribution of myosin-mediated forces governs red blood cell membrane curvature through tension modulation
Source: PLoS Comput Biol. 2020 May 26;16(5):e1007890. doi: 10.1371/journal.pcbi.1007890 (PMC7274484; doi:10.1371/journal.pcbi.1007890)
Supplement: S3 Fig — (PDF) [file pcbi.1007890.s003.pdf]

Supplementary material for "Non-uniform distribution of myosin-mediated forces governs red blood cell membrane curvature through tension modulation"

H. Alimohamadi[1], A.S. Smith[2], R.B. Nowak[2], V.M. Fowler[2,3] and P. Rangamani[1]

[1]Department of Mechanical and Aerospace Engineering, University of California San Diego, California, United states of America
[2]Department of Molecular Medicine, The Scripps Research Institute, La Jolla, California, United states of America
[3]Department of Biological Sciences, University of Delaware, Newark, Delaware, Unites States of America

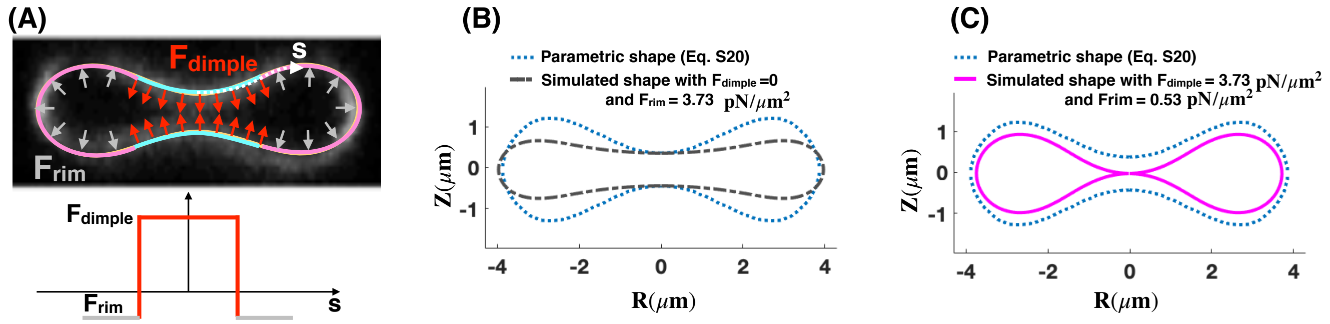

Figure S3: Applying a pushing force at the rim region increases the total shape error (Eq. S23). (A) (A, upper) Schematic of a biconcave RBC with an inward force per unit area (red arrows) at the dimple and an outward force per unit area (gray arrows) at the rim section. (A, lower) The applied force along the membrane as a function of the arclength (Eq. S24). The simulated shape of RBC with only a pulling force at the dimple region ($F_{rim} = 3.73$ $pN/\mu m^2$). Here, the total shape error is $\epsilon_{total} \sim 12.5\%$, which is larger than even the case with the uniform force per unit area (Fig. 3, center). (C) The simulated shape of RBC with both pulling and pushing forces at the dimple and rim regions respectively ($F_{dimple} = 3.73$ $pN/\mu m^2$ and $F_{rim} = 0.53$ $pN/\mu m^2$). Adding the small pushing force at the rim region compared to Fig. 4, increased the total shape error noticeably from $\epsilon_{total} \sim 5.62\%$ to $\epsilon_{total} \sim 9.71\%$.
