# Supplementary material for: Non-uniform distribution of myosin-mediated forces governs red blood cell membrane curvature through tension modulation
Source: PLoS Comput Biol. 2020 May 26;16(5):e1007890. doi: 10.1371/journal.pcbi.1007890 (PMC7274484; doi:10.1371/journal.pcbi.1007890)
Supplement: S4 Fig — (PDF) [file pcbi.1007890.s004.pdf]

# Supplementary material for “Non-uniform distribution of myosin-mediated forces governs red blood cell membrane curvature through tension modulation”

H. Alimohamadi[1], A.S. Smith[2], R.B. Nowak[2], V.M. Fowler[2,3] and P. Rangamani[1]

[1]Department of Mechanical and Aerospace Engineering, University of California San Diego, California, United states of America
[2]Department of Molecular Medicine, The Scripps Research Institute, La Jolla, California, United states of America
[3]Department of Biological Sciences, University of Delaware, Newark, Delaware, Unites States of America

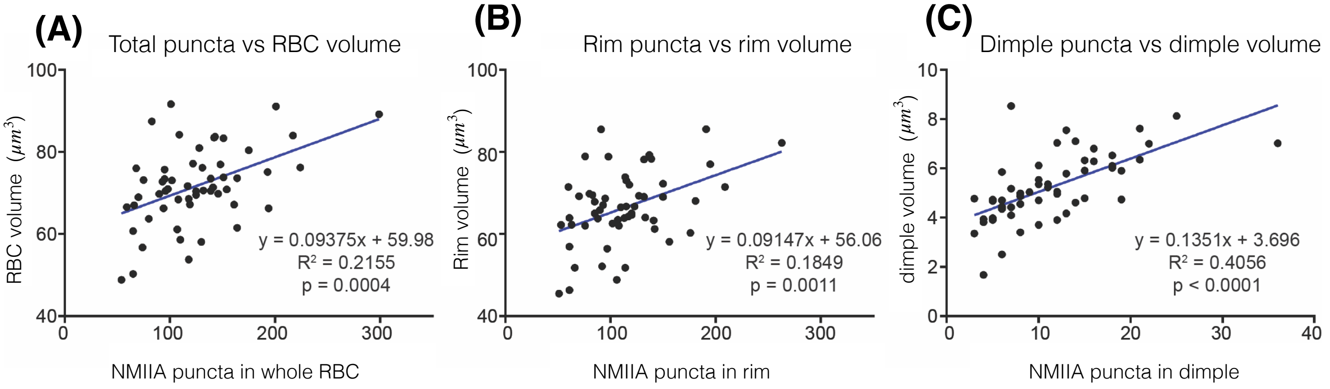


Figure S4: The number of NMIIA puncta increases with region volume. (A-C) Scatterplots of NMIIA puncta in the whole RBC versus the whole RBC volume (A), NMIIA puncta in the rim versus the rim volume (B), and NMIIA puncta in the dimple versus the dimple volume (C). Blue lines represent linear best-fit lines. The equation for the best-fit line, the $R^2$ value, and the p-value for each linear regression are given next to the chart. In all three regions, the number of NMIIA puncta increases with increasing region volume. n = 55 RBCs from 3 individual donors (same RBCs as in Fig. 5).
