# Supplementary material for: Non-uniform distribution of myosin-mediated forces governs red blood cell membrane curvature through tension modulation
Source: PLoS Comput Biol. 2020 May 26;16(5):e1007890. doi: 10.1371/journal.pcbi.1007890 (PMC7274484; doi:10.1371/journal.pcbi.1007890)
Supplement: S5 Fig — (PDF) [file pcbi.1007890.s005.pdf]

Supplementary material for “Non-uniform distribution of myosin-mediated forces governs red blood cell membrane curvature through tension modulation”

H. Alimohamadi[1], A.S. Smith[2], R.B. Nowak[2], V.M. Fowler[2,3] and P. Rangamani[1]

[1]Department of Mechanical and Aerospace Engineering, University of California San Diego, California, United states of America
[2]Department of Molecular Medicine, The Scripps Research Institute, La Jolla, California, United states of America
[3]Department of Biological Sciences, University of Delaware, Newark, Delaware, Unites States of America

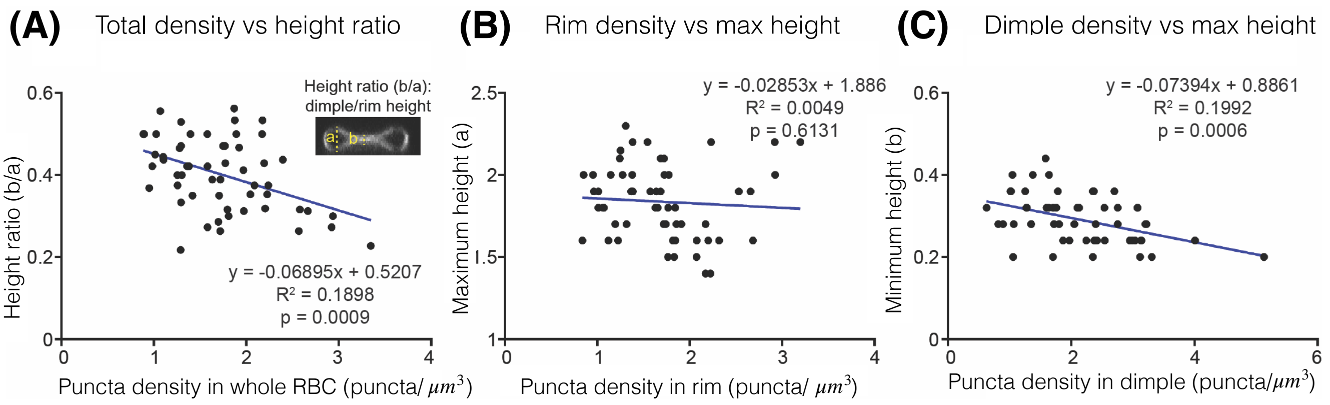

Figure S5: RBCs with higher biconcavity have higher NMIIA density in the whole RBC and in the dimple. (A-C) Predictions about the relationship between NMIIA density and RBC shape based on the model. (A) Scatterplot of NMIIA puncta density in the whole RBC versus RBC biconcavity (height ratio) measured from the ratio of cell height at the dimple to maximum cell height at the rim from an XZ slice near the center of the cell (see inset). (B) Scatterplot of NMIIA puncta density in rim region versus maximum height at the rim (rim height (a) in (A)). (C) Scatterplot of NMIIA puncta density in dimple region versus minimum height at the dimple (dimple height (b) in (A)). (A-C) Blue lines represent linear best-fit lines. The equation for the best-fit line, the R^2 value, and the p-value for each linear regression are given next to the chart. Height ratio generally decreases (indicating increasing biconcavity) with increasing whole RBC puncta density (A), and minimum height at the dimple generally decreases with increasing dimple puncta density (C), though there is high variability in this trend between individual cells. Maximum height at the rim does not correlate with rim puncta density (B). n = 55 RBCs from 3 individual donors (same RBCs as in Fig. 5).
