# Supplementary material for: Non-uniform distribution of myosin-mediated forces governs red blood cell membrane curvature through tension modulation
Source: PLoS Comput Biol. 2020 May 26;16(5):e1007890. doi: 10.1371/journal.pcbi.1007890 (PMC7274484; doi:10.1371/journal.pcbi.1007890)
Supplement: S6 Fig — (PDF) [file pcbi.1007890.s006.pdf]

Supplementary material for "Non-uniform distribution of myosin-mediated forces governs red blood cell membrane curvature through tension modulation"

H. Alimohamadi[1], A.S. Smith[2], R.B. Nowak[2], V.M. Fowler[2,3] and P. Rangamani[1]

[1]Department of Mechanical and Aerospace Engineering, University of California San Diego, California, United states of America
[2]Department of Molecular Medicine, The Scripps Research Institute, La Jolla, California, United states of America
[3]Department of Biological Sciences, University of Delaware, Newark, Delaware, Unites States of America

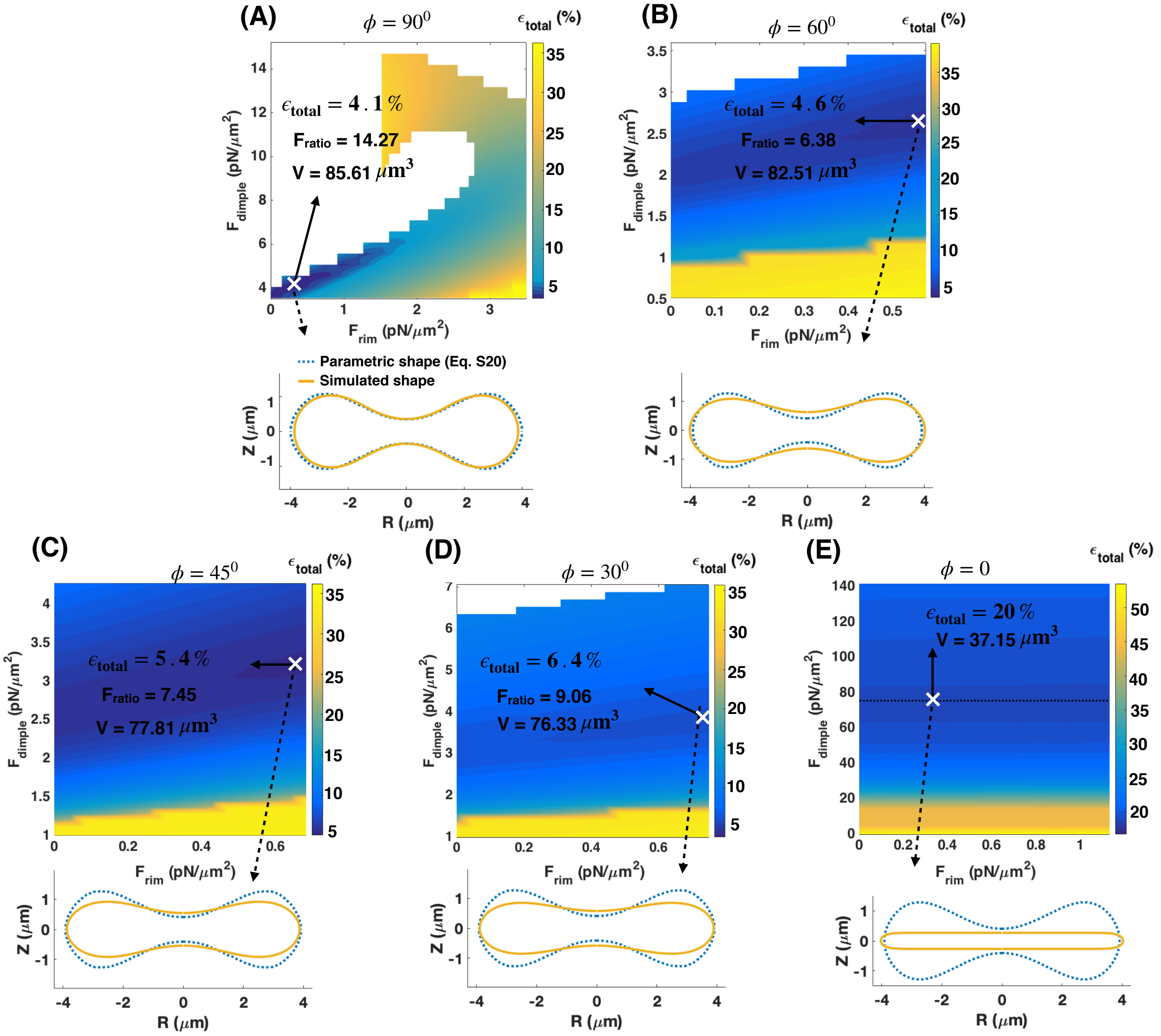

Figure S6: For tensionless membrane (Tension =0), deviation of the applied forces from normal ($\phi = 90^0$) to the tangential orientation ($\phi = 0$) results in the formation of pancake-shaped geometries with large shape error. The Heat maps show the total error in the shape of the simulated RBCs for a range of force densities in the dimple and rim regions. (A) The applied forces are assumed to be normal ($\phi = 90^0$). (B) The applied forces make angle $\phi = 60^0$ with the tangent vector $\mathbf{a}_s$. (C) The applied forces make angle $\phi = 45^0$ with the tangent vector $\mathbf{a}_s$. (D) The applied forces make angle $\phi = 30^0$ with the tangent vector $\mathbf{a}_s$. (E) The applied forces are tangent to the membrane surface ($\phi = 0$). In each heat map, the point with the minimum error is marked with '**X**'. Also, for each marked point, the volume of the simulated RBC (V) is calculated and the shape (solid yellow line) is shown in comparison with the reference parametric (dotted blue line). In the case of tangential forces ($\phi = 0$), the shape error is independent of the magnitude of the rim force density (F_{rim}).
