# Supplementary material for: Non-uniform distribution of myosin-mediated forces governs red blood cell membrane curvature through tension modulation
Source: PLoS Comput Biol. 2020 May 26;16(5):e1007890. doi: 10.1371/journal.pcbi.1007890 (PMC7274484; doi:10.1371/journal.pcbi.1007890)
Supplement: S9 Fig — (PDF) [file pcbi.1007890.s009.pdf]

Supplementary material for "Non-uniform distribution of myosin-mediated forces governs red blood cell membrane curvature through tension modulation"

H. Alimohamadi[1], A.S. Smith[2], R.B. Nowak[2], V.M. Fowler[2,3] and P. Rangamani[1]

[1]Department of Mechanical and Aerospace Engineering, University of California San Diego, California, United states of America
[2]Department of Molecular Medicine, The Scripps Research Institute, La Jolla, California, United states of America
[3]Department of Biological Sciences, University of Delaware, Newark, Delaware, Unites States of America

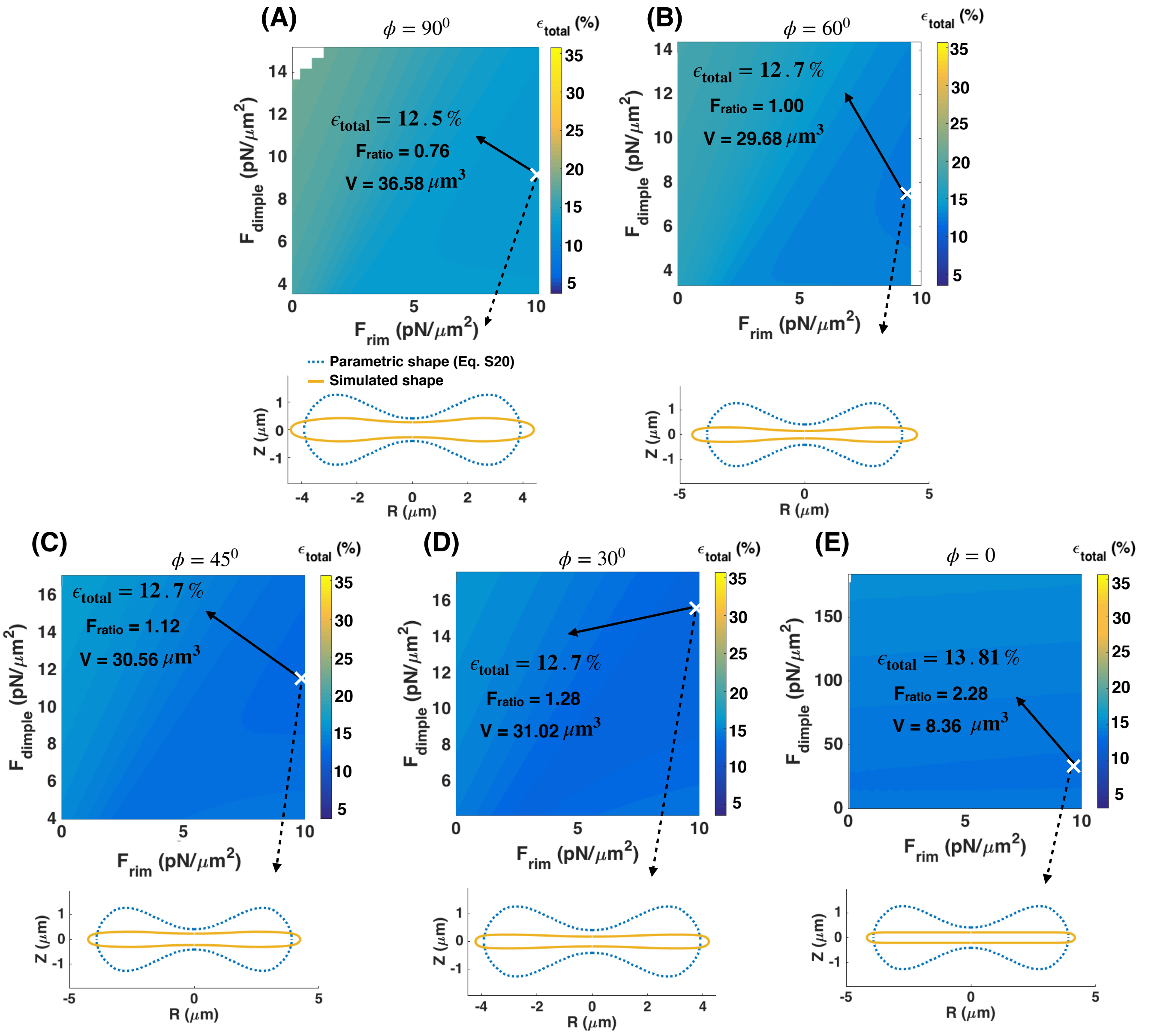

Figure S9: For high membrane tension (Tension $=10^{-2}$ pN/nm), independent of the orientation of the force, the simulated shapes have pancake morphologies and the shape error is too large ($\epsilon_{\text{total}} > 0.5\ \mu$m). The Heat maps show the total error in the shape of the simulated RBCs for a range of force densities in the dimple and rim regions. (A) The applied forces are assumed to be normal ($\phi = 90^0$). (B) The applied forces make angle $\phi = 60^0$ with the tangent vector $\mathbf{a}_s$. (C) The applied forces make angle $\phi = 45^0$ with the tangent vector $\mathbf{a}_s$. (D) The applied forces make angle $\phi = 30^0$ with the tangent vector $\mathbf{a}_s$. (E) The applied forces are tangent to the membrane surface ($\phi = 0$). In each heat map, the point with the minimum error is marked with '**X**'. Also, for each marked point, the volume of the simulated RBC (V) is calculated and the shape (solid yellow line) is shown in comparison with the reference parametric .
