# Supplementary material for: Non-uniform distribution of myosin-mediated forces governs red blood cell membrane curvature through tension modulation
Source: PLoS Comput Biol. 2020 May 26;16(5):e1007890. doi: 10.1371/journal.pcbi.1007890 (PMC7274484; doi:10.1371/journal.pcbi.1007890)
Supplement: S1 Table — (PDF) [file pcbi.1007890.s010.pdf]

Supplementary material for "Non-uniform distribution of myosin-mediated forces governs red blood cell membrane curvature through tension modulation"

H. Alimohamadi[1], A.S. Smith[2], R.B. Nowak[2], V.M. Fowler[2,3] and P. Rangamani[1]

[1]Department of Mechanical and Aerospace Engineering, University of California San Diego, California, United States of America
[2]Department of Molecular Medicine, The Scripps Research Institute, La Jolla, California, Unites States of America
[3]Department of Biological Sciences, University of Delaware, Newark, Delaware, United States of America

Table 1: Notation used in the model

Notation	Description	Units
p	Pressure difference across the membrane	pN/nm^2
θ^α	The surface coordinate ($\alpha \in \{1,2\}$)	
W	Local energy per unit area	pN/nm
E	Total surface energy	pN·nm
E_b	Bending energy	pN·nm
E_f	Work done by forces	pN·nm
$\mathbf{r}$	Position vector	
$\mathbf{n}$	Normal unit vector to the membrane surface	unit vector
$\mathbf{a_s}$	Tangent unit vector on the membrane surface	unit vector
$\boldsymbol{\nu}$	Tangent vector to the membrane surface in direction of increasing arclength	unit vector
$\boldsymbol{\tau}$	Rightward normal in direction of revolution	unit vector
$\mathbf{a}_\xi$	Basis vectors describing the tangent plane	
λ	Membrane tension	pN/nm
H	Mean curvature of the membrane	1/nm
K	Gaussian curvature of the membrane	$1/\mathrm{nm}^2$
F	Normally applied force per unit area	pN/$\mu\mathrm{m}^2$
κ_ν	Tangential curvature	1/nm
κ_τ	Transverse curvature	1/nm
κ	Bending modulus	pN · nm
κ_G	Gaussian modulus	pN · nm
s	Arclength	nm
θ	Azimuthal angle	
ψ	Angle between $\mathbf{e}_r$ and $\mathbf{a}_s$	
R	Radial distance	nm
Z	Elevation from base plane	nm
$\mathbf{e}_r(\theta)$	Radial basis vector	unit vector
$\mathbf{e}_\theta$	Azimuthal basis vector	unit vector
$\mathbf{k}$	Altitudinal basis vector	unit vector
A	Membrane area	nm^2
s_{max}	Maximum arclength	nm
$\mathrm{h}_{\mathrm{max}}$	Maximum height at the rim	
$\mathrm{h}_{\mathrm{min}}$	Minimum height at the dimple	
L	The maximum cell diameter	
ϵ_{hmax}	Error in the maximum height	
ϵ_{hmin}	Error in the minimum height	
ϵ_{L}	Error in the length	
$\epsilon_{\mathrm{total}}$	Total error	
M	Shape equation variable	1/nm
