# Supplementary material for: Non-uniform distribution of myosin-mediated forces governs red blood cell membrane curvature through tension modulation
Source: PLoS Comput Biol. 2020 May 26;16(5):e1007890. doi: 10.1371/journal.pcbi.1007890 (PMC7274484; doi:10.1371/journal.pcbi.1007890)
Supplement: S2 Table — (PDF) [file pcbi.1007890.s011.pdf]

Supplementary material for “Non-uniform distribution of myosin-mediated forces governs red blood cell membrane curvature through tension modulation”

H. Alimohamadi[1], A.S. Smith[2], R.B. Nowak[2], V.M. Fowler[2,3] and P. Rangamani[1]

[1]Department of Mechanical and Aerospace Engineering, University of California San Diego, California, United States of America
[2]Department of Molecular Medicine, The Scripps Research Institute, La Jolla, California, United States of America
[3]Department of Biological Sciences, University of Delaware, Newark, Delaware, United States of America

Table 2: Notation used in the model

Notation	Description	Units
R_0	Radius of the RBC cell	nm
κ_0	Bending rigidity of the lipid	pN
F_{uniform}	Uniformly applied force	$\text{pN}/\mu\text{m}^2$
F_{dimple}	Local force at dimple region	$\text{pN}/\mu\text{m}^2$
F_{rim}	Local force at rim region	$\text{pN}/\mu\text{m}^2$
A_{dimple}	Membrane surface are in the dimple region	m^2
A_{rim}	Membrane surface are in the rim region	m^2
V_{dimple}	Occupied volume by the dimple region	m^3
V_{rim}	Occupied volume by the rim region	m^3
r	Dimensionless radial distance	
z	Dimensionless height	
h	Dimensionless mean curvature	
m	Dimensionless M	
$\tilde{\lambda}$	Dimensionless surface tension	
$\tilde{p}$	Dimensionless pressure	
$\tilde{\mathbf{f}}$	Dimensionless force	
$\tilde{\kappa}$	Dimensionless bending modulus	
t	Dimensionless arclength	
F_{ratio}	Ratio of the force at the dimple versus the rim region	
