# Supplementary material for: Non-uniform distribution of myosin-mediated forces governs red blood cell membrane curvature through tension modulation
Source: PLoS Comput Biol. 2020 May 26;16(5):e1007890. doi: 10.1371/journal.pcbi.1007890 (PMC7274484; doi:10.1371/journal.pcbi.1007890)
Supplement: S1 Text — (PDF) [file pcbi.1007890.s012.pdf]

Supplementary material for "Non-uniform distribution of myosin-mediated forces governs red blood cell membrane curvature through tension modulation"

H. Alimohamadi[1], A.S. Smith[2], R.B. Nowak[2], V.M. Fowler[2,3] and P. Rangamani[1]

[1]Department of Mechanical and Aerospace Engineering, University of California San Diego, California, United states of America
[2]Department of Molecular Medicine, The Scripps Research Institute, La Jolla, California, United states of America
[3]Department of Biological Sciences, University of Delaware, Newark, Delaware, Unites States of America

1 Model description

1.1 Assumptions

- We consider that the radii of the membrane curvatures are much larger than the thickness of the bilayer [1]. This allows us to treat the lipid bilayer as a thin elastic shell and model the bending energy of the membrane by the Helfrich–Canham energy, which depends only on the local curvatures of the surface and compositional heterogeneities [2, 3].
- Due to the high stretching modulus of lipid bilayers, we assume that the membrane is locally incompressible [4]. We use a Lagrange multiplier to implement this constraint [5–7].
- We assume that the RBC is at mechanical equilibrium at all times, allowing us to neglect inertia [8–10]. This assumption is consistent with the experimentally observed shapes for the resting RBCs in both *vivo* and *vitro* [11, 12].
- We assume that the total surface area of the RBC membrane is constant and is $135\ \mu\text{m}^2$ [13, 14]. All our simulations are conducted using this constant area.
- For simplicity in the numerical simulations, we assume that the RBC is rotationally symmetric and also has a reflection symmetry with respect to the Z = 0 plane (see Fig. 1C) [3, 13, 15, 16]. This assumption reduces the computational cost of the simulation to simply calculating the shape of the curve shown by red dotted line in Fig. 1C.

1.2 Membrane mechanics

In this section, we present a concise derivation of the governing mathematical shape equations for the RBC membrane at mechanical equilibrium. The complete derivation with details are given in [5, 17–20].
The total free energy of the lipid bilayer (E) including the bending energy of the membrane (E_b) and the work done by the applied forces by the membrane skeleton (E_f) is given by [20–23]

$$E = E_b - E_f, \tag{S1}$$

where

$$E_b = \int_\omega (W(H,K;\theta^\alpha) + \lambda(\theta^\alpha))da - pV, \quad \text{and} \tag{S2a}$$

$$E_f = \int_\omega \mathbf{F}(\theta^\alpha).(\mathbf{r} - \mathbf{r}_0)da. \tag{S2b}$$

Here ω is the total membrane surface area, W is the energy density, θ^α denotes the surface coordinate where $\alpha \in \{1,2\}$, H is the mean curvature of the surface, K is the Gaussian curvature, λ is the membrane tension field which is the Lagrange multiplier associated with the local area constraint, p is is the transmembrane pressure which is the Lagrange multiplier associated with the volume constraint, V is the enclosed volume, $\mathbf{F}$ is the force per unit area, $\mathbf{r}$ is the position vector in the current configuration, and $\mathbf{r}_0$ is the position vector in the reference frame.

Substituting Eq.s S2a and S2b into Eq. S1 and using the variational approach to minimize the total energy gives us the so-called "shape equation" and the incompressibility condition [5, 18, 20]

$$\Delta\frac{1}{2}W_H + W_H(2H^2 - K) + 2H(KW_K - W) - 2\lambda H = p + \mathbf{F}\cdot\mathbf{n}, \tag{S3a}$$

$$\lambda_{,\alpha} + \frac{\partial W}{\partial x^\alpha_{|\text{exp}}} = -\mathbf{F}\cdot\mathbf{a}_s, \tag{S3b}$$

where $\Delta(\cdot) = (\cdot)_{;\alpha\beta}a^{\alpha\beta}$ is the surface Laplacian where $(.)_{;\alpha}$ denotes the covariant derivative and $a^{\alpha beta}$ is the dual metric, $\mathbf{n}$ is the unit normal vector to the membrane surface, $\mathbf{a}_\alpha$ is the unit tangent vector in the α direction, and $(.)_{|\text{exp}}$ represents the explicit derivative with respect to coordinate θ^α.

1.3 Helfrich-Canham energy

In this study, to model the bending energy of the RBC membrane, we used the classical Helfrich-Canham energy given by [1, 2, 23]

$$W(H,K;\theta^\alpha) = \kappa H(\theta^\alpha)^2 + \kappa_G K(\theta^\alpha), \tag{S4}$$

where κ and κ_G are constants representing the bending and Gaussian moduli respectively.

Using Helfrich-Canham energy (Eq. S4) simplifies the shape equation (Eq. S3a) and the incompressibility condition (Eq. S3b) as

$$\kappa\Delta H + 2\kappa H(H^2 - K) = p + 2\lambda H + \mathbf{F}\cdot\mathbf{n}, \tag{S5a}$$

$$\lambda_{,\alpha} = -\mathbf{F}\cdot\mathbf{a}_\alpha, \tag{S5b}$$

where $(.)_{,\alpha}$ is the partial derivative with respect to the coordinate θ^α.

1.4 Governing equations in axisymmetric coordinates

We assumed the RBC has a rotationally symmetric shape and define the surface of revolution (Fig. 1C) by

$$\mathbf{r}(s,\theta) = R(s)\mathbf{e}_r(\theta) + Z(s)\mathbf{k}, \tag{S6}$$

where s is the arclength along the curve, R(s) is the radius from the axis of rotation, Z(s) is the height from the base plane, and $(\mathbf{e}_r, \mathbf{e}_\theta, \mathbf{k})$ form the basis coordinate. Defining ψ as the angle made by the tangent with respect to the vertical gives

$$R'(s) = \cos(\psi), \quad Z'(s) = \sin(\psi), \tag{S7}$$

which satisfies the identity $(R')^2 + (Z')^2 = 1$, where $()'$ is the partial derivative with respect to the arclength. Using Eq. S7, we can define the normal ($\mathbf{n}$) and tangent ($\mathbf{a}_s$) vectors to the surface as

$$\mathbf{n} = -\sin\psi \mathbf{e}_r(\theta) + \cos\psi \mathbf{k}, \quad \mathbf{a}_s = \cos\psi \mathbf{e}_r(\theta) + \sin\psi \mathbf{k}. \tag{S8}$$

This parameterization allows us to write the tangential (κ_ν) and transverse (κ_τ) curvatures as

$$\kappa_\nu = \psi', \quad \kappa_\tau = \frac{\sin\psi}{R}. \tag{S9}$$

The mean curvature (H) and Gaussian curvature (K) are obtained by summation and multiplication of the tangential and transverse curvatures

$$H = \frac{1}{2}(\kappa_\nu + \kappa_\tau) = \frac{1}{2}(\psi' + \frac{\sin\psi}{R}), \quad K = \kappa_\tau \kappa_\nu = \frac{\psi' \sin\psi}{R}. \tag{S10}$$

Finally, we define $M = \frac{1}{2\kappa} R (W_H)'$ to reduce the governing equations (Eq. S5a and Eq. S5b) to a system of first- order differential equations with six unknowns R, Z, ψ, H, M, and λ [17, 24, 25],

$$\begin{gathered} R' = \cos\psi, \quad Z' = \sin\psi, \quad R\psi' = 2RH - \sin\psi, \quad RH' = M, \\ \frac{M'}{R} = \frac{p}{\kappa} + \frac{\mathbf{F}\cdot\mathbf{n}}{\kappa} + \frac{2H\lambda}{\kappa} - 2H\left(H - \frac{\sin\psi}{R}\right)^2 \\ \text{and } \lambda' = -\mathbf{F}\cdot\mathbf{a}_s. \end{gathered} \tag{S11}$$

In order to solve the system of equations in Eq. S11, we need to provide six boundary conditions. We consider an axisymmetric RBC with reflection symmetry with respect to the Z = 0 plane (see Fig. 1C). These assumptions can be applied as the following boundary conditions,

$$\begin{gathered} R(0^+) = 0, \quad \psi(0^+) = 0, \quad Z(\mathrm{s}_{\max}) = 0, \\ \psi(\mathrm{s}_{\max}) = \frac{-\pi}{2}, \quad M(\mathrm{s}_{\max}) = 0, \quad \text{and} \quad \lambda(\mathrm{s}_{\max}) = \lambda_0, \end{gathered} \tag{S12}$$

where $\mathrm{s}_{\max}$ is the maximum length of the computational domain and λ_0 is the prescribed membrane tension. One advantage of an asymmetric coordinate system is that the manifold area (A) and the occupied volume (V) can be expressed in term of arclength,

$$A(s) = 2\pi \int_0^s R(\eta) d\eta, \tag{S13a}$$

$$V(s) = 2\pi \int_0^s R(\eta) Z(\eta) \cos(\psi) d\eta, \tag{S13b}$$

which allows us to conserve the total area of the RBC by changing the maximum length of the computational domain ($\mathrm{s}_{\max}$).

Considering the spherical shape of RBC with no applied force, we can calculate the reduced volume (v) as

$$v = V/V_{\text{sphere}} \tag{S14}$$

where V_{sphere} is the volume of the sphere that we get from simulation when there is no applied force along membrane ($F = 0$).

1.5 Nondimensionalization

In order to perform the numerical computations, we nondimensionalized the system of equations (Eq. S11) by using two positive constants, the radius of the RBC (R_0) and the lipid bilayer bending rigidity (κ_0). This allows us to define the dimensionless variables as

$$\begin{gathered} t = \frac{s}{R_0}, \quad r = \frac{R}{R_0}, \quad z = \frac{Z}{R_0}, \quad h = HR_0 \quad m = MR_0 \\ \tilde{\lambda} = \frac{\lambda R_0^2}{\kappa_0}, \quad \tilde{p} = \frac{pR_0^3}{\kappa_0} \quad \mathbf{f} = \frac{\mathbf{F}R_0^3}{\kappa_0}, \quad \tilde{\kappa} = \frac{\kappa}{\kappa_0}. \end{gathered} \tag{S15}$$

Rewriting Eq. S11 in terms of the dimensionless variables, we get [25]

$$\begin{gathered} \dot{r} = \cos\psi, \quad \dot{z} = \sin\psi, \quad r\dot{\psi} = 2rh - \sin\psi, \quad x\dot{h} = m, \\ \frac{\dot{m}}{r} = \frac{\tilde{p}}{\tilde{\kappa}} + \frac{\mathbf{f}\cdot\mathbf{n}}{\tilde{\kappa}} + \frac{2h\tilde{\lambda}}{\tilde{\kappa}} - 2h\left(h - \frac{\sin\psi}{r}\right)^2, \quad \dot{\tilde{\lambda}} = -\mathbf{f}\cdot\mathbf{a}_s, \end{gathered} \tag{S16}$$

where ($\dot{}$) is the partial derivative with respect to t. With the defined dimensionless variables in Eq. S15, the boundary conditions simplified as

$$\begin{gathered} r(0^+) = 0, \quad \psi(0^+) = 0, \quad z(\mathrm{t_{max}}) = 0 \\ \psi(\mathrm{t_{max}}) = \frac{-\pi}{2}, \quad m(\mathrm{t_{max}}) = 0, \quad \text{and} \quad \tilde{\lambda}(\mathrm{t_{max}}) = \tilde{\lambda}_0. \end{gathered} \tag{S17}$$

1.6 Parametrization of RBC biconcave morphology and shape error estimation

There are several parametric models to describe the biconcave morphology of an RBC [26–29]. Initially, Funaki proposed the Cassini oval model for the RBC biconcave morphology given by [26]

$$(R^2 + Z^2 + a^2) - 4a^2R^2 = c^4, \tag{S18}$$

where a and c are constants with the condition that $a < c < \sqrt{2}a$. Yurkin modified the Cassini oval model to an implicit equation with four constants given by [27]

$$R^4 + 2C_4R^2Z^2 + Z^4 + C_1R^2 + C_2Z^2 + C_3 = 0, \tag{S19}$$

where C_1, C_2, C_3, and C_4 are constants that depended on the RBC dimension ($C_1 = -14.85,\ C_2 = 40.40,\ C_3 = -6.65$, and $C_4 = -0.30$). Borovoi et al. introduced a function that represents the biconcave shape of an RBC in the spherical coordinate as [28]

$$R(\theta) = e\sin^q(\theta) + b, \tag{S20}$$

where e, b, and q are constants that were determined by fitting the function to the standard shape of an RBC ($e = 3$ μm, $b = 0.75$ μm, and $q = 5$). The most realistic model for the biconcave shape of an RBC was proposed by Evans and Fung based on direct experimental measurements [29]

$$Z(R) = \pm 0.5\sqrt{1 - (\frac{2R}{L})}(D_1 + D_2(\frac{2R}{L})^2 - D_3(\frac{2R}{L})^4), \tag{S21}$$

where D_1, D_2, and D_3 are the constants that were calculated by fitting the function to the average dimension of an experimentally observed RBC and statistical analysis ($D_1 = 0.81\ \mu\text{m}$, $D_2 = 7.83\ \mu\text{m}$, and $D_3 = -4.39\ \mu\text{m}$). We plotted these different proposed parametric models together for the biconcave shape of an RBC for fixed h_{min}, h_{max}, and L (Fig. 2B). In this study, we used the given parametric function by Evans and Fung (Eq. S21) as the reference geometry for the RBC experimental shape.

Typically, there is a mismatch between each RBC shape obtained from our mechanical model (Eq. S16) and the RBC parametric shape (Eq. S21). Considering the three characteristic lengths (h_{min}, h_{max}, and L in Fig. 1C), we can define three errors that characterize the difference between each of these lengths in the simulated shapes and the reference experimental shape,

$$\begin{aligned} \epsilon_{\text{hmax}} &= \frac{|\text{h}_{\text{max,par}} - \text{h}_{\text{max,sim}}|}{\text{L}_{\text{par}}} = \frac{|\Delta \text{h}_{\text{max}}|}{\text{L}_{\text{par}}} \\ \epsilon_{\text{hmin}} &= \frac{|\text{h}_{\text{min,par}} - \text{h}_{\text{min,sim}}|}{\text{L}_{\text{par}}} = \frac{|\Delta \text{h}_{\text{min}}|}{\text{L}_{\text{par}}} \\ \epsilon_{\text{L}} &= \frac{|\text{L}_{\text{par}} - \text{L}_{\text{sim}}|}{\text{L}_{\text{par}}} = \frac{|\Delta \text{L}|}{\text{L}_{\text{par}}}, \end{aligned} \tag{S22}$$

where $(.)_{\text{sim}}$ is the calculated length from the simulated shape and $(.)_{\text{par}}$ is the measured length in the parametric RBC shape (Eq. S21). The total error (ϵ_{total}) in the shape of the simulated RBC can be calculated by the root mean square (RMS) between each two mapped points of the simulated and parametric shapes

$$\epsilon_{\text{total}} = \frac{\sqrt{\frac{1}{N}\left[\sum_{i=1}^{i=N}(Z_{i,\text{sim}} - Z_{i,\text{par}})^2 + (R_{i,\text{sim}} - R_{i,\text{par}})^2\right]}}{\text{L}_{\text{par}}}, \tag{S23}$$

where N is the total number of nodes across the RBC shapes, i is the index node, $Z_{i,\text{sim}}$ and $Z_{i,\text{par}}$ are the height of the simulated and the RBC parametric (Eq. 3) shapes at index i, respectively. $R_{i,\text{sim}}$ is the radius of the simulated shape at index i, and $R_{i,\text{par}}$ is the radius of the RBC parametric shape (Eq. 3) at index i (see Fig. 2C). To trace the simulated shape and the parametric shape (Eq. 3), we mapped the points with the maximum height, minimum height, and maximum radius. Then, we discretized the domain equally between the points and calculated the errors.

1.7 Numerical implementation

We solved the system of first-order differential equations (Eq. S16) with boundary conditions Eq. S17 by the finite element software COMSOL MULTIPHYSICS ® 5.3a, using the "General Form PDE" module. Here, we summarize the steps and assumptions that we used for each simulation.

- All the simulations were performed for fixed total arclength. However, in each simulation, the maximum arclength (s_{max}) varies to conserve the total area of the RBC membrane.
- The computational domain (t) was discretized equally with mesh size = 0.001.
- To have a sharp but smooth transition in the distribution of the force ($\mathbf{f}$), we used a hyperbolic tangent function given by

$$\mathbf{f} = \frac{1}{2}[\tanh(g(t - s_{\text{dimple}}))], \tag{S24}$$

  where g is a constant (here we set g=20) and s_{dimple} represents the length scale that the local force at the dimple is applied.
- The applied force in Figs. 3-5 and Fig. 7-8 was progressively increased such that each solution was used as an initial guess for the next step.

References

1. H. Deuling and W. Helfrich, "Red blood cell shapes as explained on the basis of curvature elasticity," *Biophysical journal*, vol. 16, no. 8, pp. 861–868, 1976.

2. W. Helfrich, "Elastic properties of lipid bilayers: theory and possible experiments," *Zeitschrift fur Naturforschung C*, vol. 28, no. 11-12, pp. 693–703, 1973.

3. P. B. Canham, "The minimum energy of bending as a possible explanation of the biconcave shape of the human red blood cell," *Journal of theoretical biology*, vol. 26, no. 1, pp. 61–81, 1970.

4. W. Rawicz, K. Olbrich, T. McIntosh, D. Needham, and E. Evans, "Effect of chain length and unsaturation on elasticity of lipid bilayers," *Biophysical journal*, vol. 79, no. 1, pp. 328–339, 2000.

5. D. Steigmann, "Fluid films with curvature elasticity," *Archive for Rational Mechanics and Analysis*, vol. 150, no. 2, pp. 127–152, 1999.

6. P. Rangamani, A. Agrawal, K. K. Mandadapu, G. Oster, and D. J. Steigmann, "Interaction between surface shape and intra-surface viscous flow on lipid membranes," *Biomechanics and modeling in mechanobiology*, pp. 1–13, 2013.

7. H. Alimohamadi, B. Ovryn, and P. Rangamani, "Modeling membrane nanotube morphology: the role of heterogeneity in composition and material properties," *bioRxiv*, p. 373811, 2018.

8. J. T. Jenkins, "The equations of mechanical equilibrium of a model membrane," *SIAM Journal on Applied Mathematics*, vol. 32, no. 4, pp. 755–764, 1977.

9. J. Jenkins, "Static equilibrium configurations of a model red blood cell," *Journal of mathematical biology*, vol. 4, no. 2, pp. 149–169, 1977.

10. B. K. Pai and H. D. Weymann, "Equilibrium shapes of red blood cells in osmotic swelling," *Journal of biomechanics*, vol. 13, no. 2, pp. 105–112, 1980.

11. G. Lim HW, M. Wortis, and R. Mukhopadhyay, "Red blood cell shapes and shape transformations: newtonian mechanics of a composite membrane: sections 2.1–2.4," *Soft Matter: Lipid Bilayers and Red Blood Cells*, vol. 4, pp. 83–139, 2008.

12. N. Mohandas and J. Chasis, "Red blood cell deformability, membrane material properties and shape: regulation by transmembrane, skeletal and cytosolic proteins and lipids.," in *Seminars in hematology*, vol. 30, pp. 171–192, 1993.

13. Y. Fung and P. Tong, "Theory of the sphering of red blood cells," *Biophysical journal*, vol. 8, no. 2, pp. 175–198, 1968.

14. H. Park, S. Lee, M. Ji, K. Kim, Y. Son, S. Jang, and Y. Park, "Measuring cell surface area and deformability of individual human red blood cells over blood storage using quantitative phase imaging," *Scientific reports*, vol. 6, p. 34257, 2016.

15. J. C. Luke, "A method for the calculation of vesicle shapes," *SIAM Journal on Applied Mathematics*, vol. 42, no. 2, pp. 333–345, 1982.

16. S. Svetina and B. Žekš, "Membrane bending energy and shape determination of phospholipid vesicles and red blood cells," *European biophysics journal*, vol. 17, no. 2, pp. 101–111, 1989.

17. A. Agrawal and D. J. Steigmann, "Modeling protein-mediated morphology in biomembranes," *Biomechanics and modeling in mechanobiology*, vol. 8, no. 5, pp. 371–379, 2009.

18. P. Rangamani, K. K. Mandadap, and G. Oster, “Protein-induced membrane curvature alters local membrane tension,” *Biophysical journal*, vol. 107, no. 3, pp. 751–762, 2014.

19. H. Alimohamadi, B. Ovryn, and P. Rangamani, “Protein aggregation and membrane bending govern nanotube morphology.,” *bioRxiv*, p. 373811, 2018.

20. N. Walani, J. Torres, and A. Agrawal, “Endocytic proteins drive vesicle growth via instability in high membrane tension environment,” *Proceedings of the National Academy of Sciences*, vol. 112, no. 12, pp. E1423–E1432, 2015.

21. N. Walani, J. Torres, and A. Agrawal, “Anisotropic spontaneous curvatures in lipid membranes,” *Physical Review E*, vol. 89, no. 6, p. 062715, 2014.

22. M. Lokar, D. Kabaso, N. Resnik, K. Sepčić, V. Kralj-Iglič, P. Veranič, R. Zorec, and A. Iglič, “The role of cholesterol-sphingomyelin membrane nanodomains in the stability of intercellular membrane nanotubes,” *International journal of nanomedicine*, vol. 7, p. 1891, 2012.

23. H. Alimohamadi and P. Rangamani, “Modeling membrane curvature generation due to membrane–protein interactions,” *Biomolecules*, vol. 8, no. 4, p. 120, 2018.

24. J. E. Hassinger, G. Oster, D. G. Drubin, and P. Rangamani, “Design principles for robust vesiculation in clathrin-mediated endocytosis,” *Proceedings of the National Academy of Sciences*, vol. 114, no. 7, pp. E1118–E1127, 2017.

25. H. Alimohamadi, R. Vasan, J. Hassinger, J. C. Stachowiak, and P. Rangamani, “The role of traction in membrane curvature generation,” *Mol. Biol. Cell*, vol. 29, no. 16, pp. 2024–2035, 2018.

26. H. FUNAKI, “Contributions on the shapes of red blood corpuscles,” *The Japanese journal of physiology*, vol. 5, pp. 81–92, 1955.

27. M. A. Yurkin *et al.*, *Discrete dipole simulations of light scattering by blood cells*. Universiteit van Amsterdam [Host], 2007.

28. A. G. Borovoi, E. I. Naats, and U. G. Oppel, “Scattering of light by a red blood cell,” *Journal of biomedical optics*, vol. 3, no. 3, pp. 364–373, 1998.

29. E. Evans and Y.-C. Fung, “Improved measurements of the erythrocyte geometry,” *Microvascular research*, vol. 4, no. 4, pp. 335–347, 1972.
